# HIV prevention costs and program scale: data from the PANCEA project in five low and middle-income countries

**DOI:** 10.1186/1472-6963-7-108

**Published:** 2007-07-12

**Authors:** Elliot Marseille, Lalit Dandona, Nell Marshall, Paul Gaist, Sergio Bautista-Arredondo, Brandi Rollins, Stefano M Bertozzi, Jerry Coovadia, Joseph Saba, Dmitry Lioznov, Jo-Ann Du Plessis, Evgeny Krupitsky, Nicci Stanley, Mead Over, Alena Peryshkina, SG Prem Kumar, Sowedi Muyingo, Christian Pitter, Mattias Lundberg, James G Kahn

**Affiliations:** 1 Institute of Health Policy Studies, University of California, San Francisco, USA; 2 George Institute for International Health – India, Hyderabad, India; Health Studies Area, Centre for Human Development, Administrative Staff College of India, Hyderabad, India; School of Public Health and George Institute for International Health, University of Sydney, Sydney, Australia; 3 Office of AIDS Research, National Institutes of Health, Bethesda, USA; 4 Instituto Nacional de Salud Pública, Cuernavaca, Mexico; 5 HIVAN(Centre for HIV/AIDS Networking), Durban, South Africa; 6 Axios International, Paris, France; 7 St. Petersburg Pavlov State Medical University, St. Petersburg, Russia; 8 Elizabeth Glaser Pediatric AIDS Foundation, Washington, D.C., USA; 9 Center for Global Development, Washington, D.C., USA; 10 AIDS Infoshare, Moscow, Russia; 11 George Institute for International Health – India, Hyderabad, India; Health Studies Area, Centre for Human Development, Administrative Staff College of India, Hyderabad, India; 12 Axios International, Kampala, Uganda; 13 World Bank, Washington, D.C., USA

## Abstract

**Background:**

Economic theory and limited empirical data suggest that costs per unit of HIV prevention program output (unit costs) will initially decrease as small programs expand. Unit costs may then reach a nadir and start to increase if expansion continues beyond the economically optimal size. Information on the relationship between scale and unit costs is critical to project the cost of global HIV prevention efforts and to allocate prevention resources efficiently.

**Methods:**

The "Prevent AIDS: Network for Cost-Effectiveness Analysis" (PANCEA) project collected 2003 and 2004 cost and output data from 206 HIV prevention programs of six types in five countries. The association between scale and efficiency for each intervention type was examined for each country. Our team characterized the direction, shape, and strength of this association by fitting bivariate regression lines to scatter plots of output levels and unit costs. We chose the regression forms with the highest explanatory power (R^2^).

**Results:**

Efficiency increased with scale, across all countries and interventions. This association varied within intervention and within country, in terms of the range in scale and efficiency, the best fitting regression form, and the slope of the regression. The fraction of variation in efficiency explained by scale ranged from 26% – 96%. Doubling in scale resulted in reductions in unit costs averaging 34.2% (ranging from 2.4% to 58.0%). Two regression trends, in India, suggested an inflection point beyond which unit costs increased.

**Conclusion:**

Unit costs decrease with scale across a wide range of service types and volumes. These country and intervention-specific findings can inform projections of the global cost of scaling up HIV prevention efforts.

## Background

There is wide agreement that an effective response to the global HIV epidemic requires very substantial resources. This consensus has been partially translated into increasing contributions to combat the epidemic [1]. Aggregate commitments by major donors such as the Global Fund to Fight Malaria, TB and HIV; the U.S. President's Emergency Program for AIDS Relief (PEPFAR); the European Union; and, the Gates Foundation suggest that we have entered an era in which the total funds allocated to stem the HIV epidemic may constitute a significant portion of the amount needed. Yet the imperative to spend this money efficiently can hardly be over-stated: the lives of millions depend upon how effectively available funds are allocated. Sound resource allocation and program budgeting, in turn, must rest on a foundation of robust unit cost estimates for the most important prevention modalities in key epidemic and cultural settings.

There is a substantial, growing, but still limited body of data on the costs of HIV prevention services [2-12]. These data provide a reasonable basis for estimating service costs for some intervention types in some settings, particularly in sub-Saharan Africa. However many other intervention-setting pairs remain unexamined. In addition to an insufficient number of cost data points, data have often been gathered using different data collection instruments and compiled using different methods. These data are therefore not always directly comparable.

Micro-economic theory and empirical evidence suggest that under ordinary circumstances downward sloping average total costs flatten out and eventually turn up to form a U-shaped curve [13]. While the concept of diseconomies of scale was originally developed in conjunction with the theory of the firm [14,15], the causes of scale diseconomies are not specific to the private sector. These causes include increasing costs of communication, increasing worker alienation, bureaucratic inertia, and duplication of effort. Certain inputs may become more costly too. For example, at least over the short term, a program may exhaust the available supply of lower-wage but adequately trained staff in the area, and be forced to hire staff that are more expensive. On the demand side, after the most willing and accessible clients have been served, it becomes increasingly expensive to reach and motivate the next client. Information concerning the threshold beyond which unit costs increase can help inform plans for program expansion [16]. For example, they can help to determine whether it is more efficient to cover a given area with fewer, but larger HIV prevention facilities, or with a larger number of smaller facilities.

While cost data are limited, still less is understood about how HIV program expansion affects costs and effectiveness. Among the unanswered questions, are: "How rapidly do costs decline with scale? How does the strength of the relationship between unit cost and scale vary by intervention and by country? At what service volume do unit costs start to rise again?" In the absence of data on scale effects, efforts to project resource requirements for scaled-up HIV/AIDS programs assume constant unit costs and vary this assumption in sensitivity analyses [17,18]. It is understood that the assumption of constant unit costs may result in substantial inaccuracies [19]. We are aware of only one empirical study of the relationship between scale and unit costs of an HIV prevention program. This analysis of the effect of scale on total and on unit costs in 17 sex worker programs run by non-governmental organizations in southern India found decreasing unit cost up to about 1,000 – 1,700 sex workers served annually, after which unit costs rise in a classic U-shaped curve [20]. In a 2005 review of the costs of scaling up health interventions in developing countries, the authors found that there were insufficient data to support their goal of deriving typical unit cost curves as interventions increase in scale [21].

"Prevent AIDS: Network for Cost-Effectiveness Analysis" (PANCEA) is a five-country study funded by the U.S. National Institutes of Health. It has the purpose of providing essential information and analysis for an improved allocation of HIV prevention funds in low and middle-income countries. The study includes five countries: India, Mexico, Russia, South Africa, and Uganda. The results presented here draw on PANCEA data to describe the relationship of program efficiency (unit cost) and scale (number of units of services delivered). The PANCEA analysis ultimately aims to measure cost-effectiveness, i.e., the cost per "outcome" such as HIV cases averted. The present study assesses efficiency in the production of "outputs" such as the number of clients served, that are the proximate cause of behavior change and thus of epidemic impact.

## Methods

PANCEA design and data collection methods have been detailed previously and are summarized here [22].

### Program sample

PANCEA examined multiple interventions in varied organizational settings and countries. The number of programs sampled totaled 228, of which 206 are included in this analysis of unit cost variation by scale (Table 1). Condom social marketing and school program data were excluded due to low sample size per country (0–2). We also excluded data on an intervention-country if data were obtained from fewer than five prevention sites of that type in the country.

**Table 1:** Number and type of HIV prevention sites utilized in the analysis of relationship between scale and unit costs

	**VCT**	**SW**	**STI**	**IEC**	**RR**	**MTCT**	**Total**
India	17	15	13			15	60
Mexico	18		6	22			46
Russia – Pavlov	9		6		16		31
Russia – AI	10	10			6		26
So. Africa	14	15					29
Uganda	14						14
**All countries**	**82**	**40**	**25**	**22**	**22**	**15**	**206**

***Key***– **VCT**: Programs providing voluntary counseling and testing; **SW**: Programs targeting sex workers; **STI**: Programs that focused on treatment or prevention of sexually transmitted infections; **IEC**: Information, education and communication programs; **RR**: Risk reduction programs focusing on injection drug users; **MTCT**: Programs to reduce mother-to-child-transmission of HIV.

### Data on Outputs and Costs

We developed an integrated set of data collection instruments to portray program operations with sufficient detail and flexibility to capture variation among nominally similar program types, and with sufficient standardization to permit valid comparisons across multiple programs.

PANCEA gathered information on several service ***outputs ***for each intervention type. Among all program services, the key outputs selected for examination were those thought to capture program resources as they relate to the ability to reduce HIV risk behavior and transmission. For some interventions, the key output is based on a complete unit of service, e.g., for Voluntary Counseling and Testing (VCT), this constitutes receiving the counseling and testing sequence of VCT, through post-test counseling. For other interventions, the key output is based on person-years of core services, e.g., for IDU risk reduction, receiving one clean needle a week for a year. For yet other interventions, a mix of core services is provided and we measure overall interaction intensity, e.g., for sex worker programs, the total hours of program client contact. These key outputs are used to assess program efficiency. They are listed in Table 2.

**Table 2:** Key outputs used for analysis of variation in unit cost by scale

**Intervention**	**Key output**
VCT	Clients receiving VCT counseling* & testing
SW	Hours of contact with program clients
STI	First visits for suspected STI
IDU risk reduction	Client-year of needles (weekly)
IEC	Hours of media exposure
PMTCT	Women receiving post-test counseling

* In Russia, VCT counseling is often much briefer than UNAIDS Guidelines.

We conducted a comprehensive assessment of the ***costs ***of running HIV prevention interventions. Cost instruments were extensively adapted from templates produced by others to add a time element and pre-specified inputs specific to the interventions. The instruments record data on resources used and expenditures. Cost data were collected in five standard categories: personnel (clinical and support), recurrent goods (e.g., test kits), recurrent services (e.g., utilities), capital (e.g., computers and vehicles), and building or other operating space (purchase or rental). The instruments were piloted and revised, and programmed into Excel and MS Access. We costed all donated inputs (i.e., employed economic rather than financial costing), using unit costs for inputs as determined by local price quotes.

Data collection was co-coordinated by a group at the University of California, San Francisco (UCSF), and conducted in 2003 and 2004 by local HIV research teams. These teams were selected based on a prior record of high quality data collection and analysis, ability to assemble an appropriately skilled and managed team, and network of contacts among HIV prevention agencies. In-country collaborative teams were India (Administrative Staff College of India), Mexico (Instituto Nationale de Salud Publica), Russia (Pavlov University and AIDS Infoshare); South Africa (HIVAN and Axios International), and Uganda (Axios International). Each team underwent a two-week training program, including theoretical and practical components.

Each HIV prevention program site studied required seven to twelve person-days of work, including initial and follow-up visits. All data were reviewed by the UCSF team to identify gaps and inconsistencies, which were resolved through written and telephonic queries posed to the local research teams. Data verification visits were conducted at 10 percent of sites (typically 4 per country) to verify the accuracy of PANCEA data. Half of the sites were chosen based on surprising or interesting findings. The other half were chosen randomly. During these visits, local and UCSF team members reviewed and resolved potential problems and all key output and cost data.

We used written project records when available. For example, VCT programs usually have detailed records on the specific intervention steps such as pre-test counseling and HIV tests administered. However, available documents were often incomplete or imperfectly matched to PANCEA instruments, and thus required interpretation by respondents to provide needed data. Without inclusion of these less formal data sources, many HIV prevention programs would have been excluded; we were willing to sacrifice some precision for a more inclusive portrayal of the universe of HIV prevention programs. Some programs showed an imperfect match between the number of HIV test kits acquired and the number used in a given time period. In sites that had a greater than 10% discrepancy between the number reported to have been acquired and the number of tests administered, we assigned test kit costs according to the number used augmented by 10% to account for wastage.

### Analysis

The cost of each program is defined as the sum of all inputs (resources) multiplied by the unit costs for these inputs. We relied on financial data if a market price was paid by the program, otherwise on market value. We report in detail elsewhere on the methods and results for program total costs and cost per key output, including by category of cost [10,22] The analysis here focuses on the relationship of program scale and efficiency (cost per key output).

***Scale ***is defined as the quantity of key outputs delivered during a 12-month period (the most recent full fiscal year available). The key outputs are listed in Table 2. We determined scale directly from key output data (e.g., number of individuals receiving VCT services to post-test counseling), or calculated it based on the mix of different services and the frequency, average duration, and number of participants for each (e.g., sex worker programs).

***Program cost ***is defined as the total value of resources used to deliver program services for the same 12-month time period. This is "economic cost", i.e., includes financial expenditures as well as the market value of donated or subsidized inputs (such as volunteer labor or donated test kits). Costs in local currencies were converted into US$ based on the average exchange rate in the year for which data are presented.

***Efficiency ***(or cost per key output) is defined as the program cost divided by the scale. There is no adjustment for potential savings due to HIV infections averted.

We examined the association between scale and efficiency for all programs of an intervention type within each country. We characterized the direction, shape, and strength of association by applying all of the bivariate regression forms available in MS Excel, which include linear, logarithmic, polynomial, power, and exponential. We chose and present the regression form which yielded the highest R-square values. Due to the multiplicity of relationships examined, and to maintain a focus on scale, we did not perform multi-variate regressions for this overview analysis. We graphed efficiency versus scale using ordinal and abscissa scales (linear or logarithmic) that portray the range of results for each intervention most clearly.

### Service delivery quality

Quality of services could explain a portion of the potential association between unit cost and volume. For the 86 VCT sites, we therefore performed constructed a regression model to examine the association between quality indicators and unit cost. The independent variables included features of service delivery (i.e., duration of pre-test and post-test counseling sessions, availability of supplies, length of time clients waited to see a counselor, and the turn-around time for tests results), management (i.e., use of formal service delivery protocols, staff training, extent to which operations were monitored including politeness to clients, following protocol, use of performance incentives and penalties) and evaluation (i.e., whether an independent evaluation had ever been conducted, how often and for what purposes). The main model also included dummy variables for country. In an additional model, unit costs were adjusted for purchasing power parity.

### Ethics committee approval

The University of California, San Francisco's Committee on Human Research, approved this study.

## Results

We found that efficiency increased (unit costs decreased) with scale, across all countries and interventions we examined. This association varies within intervention and country, in terms of the observed range in efficiency and scale, the type of regression equation that provides the best fit, and slope of the regression line, and the proportion of variation in efficiency explained by scale. Of the 15 country-intervention pairs studied, the regression lines for two, STI and MTCT programs in India, suggest an inflection point beyond which we expect to see an up-turn in unit costs. Of these two, only one, the regression line for MTCT programs, was statistically significant. With simple linear functions, the regression trends were downward sloping in all cases.

Table 3 summarizes the rate at which unit costs change when output levels double (from 25^th ^percentile scale) and the portion of variability in unit costs that are explained by scale (R^2^). Both unit cost declines associated with a doubling of output and R^2 ^vary dramatically between countries and between interventions. Declines for VCT programs range from 32.5% in India to 2.4% in South Africa and R^2 ^values ranged from 0.83 in India to 0.29 in Uganda. The strongest association between decrease in unit cost and doubling of scale were found in India and Russia's STI programs, although these associations were not statistically significant. All other observed associations were substantial and statistically significant except for that found in Mexico's VCT sites which had a p-value of 0.066, just exceeding the usual 0.05 p-value for statistical significance.

**Table 3:** Strength of the relationship and coefficient of determination between unit cost and program scale

	**VCT**			**SW**			**PMTCT**		
	Unit cost decrease associated with doubling of scale ^1^	p-value of regression	Coefficient of determination (R^2^)	Unit cost decrease associated with doubling of scale ^1^	p-value of regression	Coefficient of determination (R^2^)	Unit cost decrease associated with doubling of scale ^1^	p-value of regression	Coefficient of determination (R^2^)

India	32.5%	< 0.001	0.83	41.8%	< 0.001	0.88	23.0%	0.038	0.42
Mexico	15.3%	0.066	0.20						
Russia	27.2%	0.021	0.28	41.0%	< 0.001	0.84			
South Africa	2.4%	0.033	0.33	31.2%	0.015	0.38			
Uganda	27.5%	0.045	0.29						

	**STI**			**IEC**			**RR**		

India	52.4%	0.050	0.42						
Mexico	32.3%	0.038	0.70	46.3%	< 0.001	0.91			
Russia	58.0%	0.385	0.26				34.5%/47.9% ^2^	0.004/0.022	0.45/0.96^2^
South Africa									
Uganda									

1. "Doubling" is a 100% increase in output from the first quartile output value.

2. Needle exchange programs/Rehabilitation-focused programs

### Voluntary Counseling and Testing Programs

**Figure 1:**
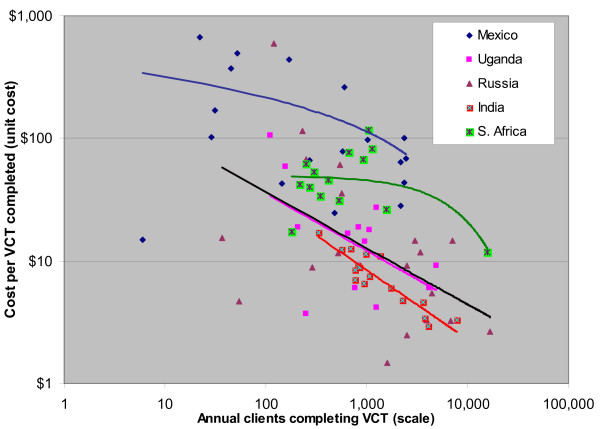


For VCT, data from all five countries show strong scale effects, i.e., sites with higher service volume tend to have lower unit costs (See Figure 1). Scale varied 100-fold within countries, and 1,000-fold across the full sample. Efficiency (cost per person receiving full VCT) varies from 10-fold to more than 100-fold within country, and over all five countries varies from $668 (in Mexico) down to $1.50 (in Russia, where counseling may be just a few minutes). The proportion of variation explained by scale varied from 20% (Mexico) to 83% (India). In Mexico, each doubling in scale is associated with a drop of $30 per VCT client (7–27%, depending on starting point). In South Africa, the effect of doubling in scale is low until more than 10,000 VCT clients (with the curve shape driven by one large program). For India, Uganda, and Russia, a doubling in size is associated with 27% to 32% lower costs.

### VCT unit cost and quality

We found no statistically significant relationship between any of our VCT program quality indicators and unit cost. The R-square for the full model, which included dummy variables for countries and adjustments for purchasing power parity was 0.05, suggesting that quality, as reflected in our analysis, explains almost none of the variation in observed unit costs.

### Sex Worker Programs

**Figure 2:**
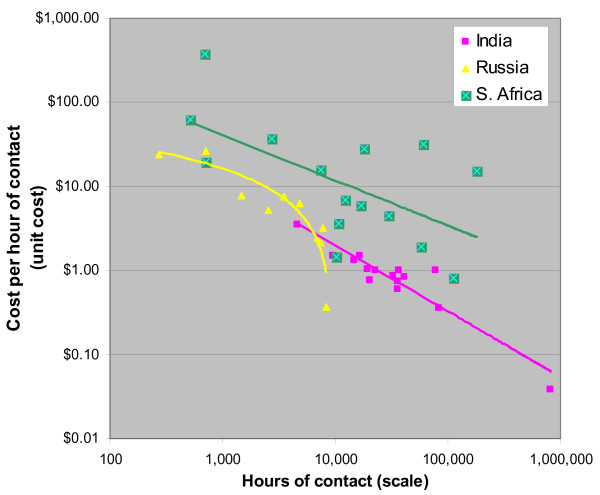


For sex worker (SW) programs, data from three countries show very strong scale effects (See Figure 2). Scale ranges 100-fold within countries, and more than 1,000-fold across the sample. Efficiency (cost per hour of contact with SWs) varies 100-fold within country, and over all three countries varies from $378 for a program in South Africa that provided only 692 hours of client contact, down to $0.04 for a program in Russia with very large group sessions, and thus many client-hours of contact. The proportion of variation explained by scale is high: 38% (South Africa), 84% (Russia), and 88% (India). All regressions were statistically significant. For South Africa, each doubling in scale is associated with a 31% decrease in cost per hour. For Russia, there is a $5 decrease per hour of contact for a doubling in scale. For India, doubling leads to a 42% drop in cost per hour. The data from India includes a program that includes large public gatherings. This generates high service volumes and very low unit costs as shown in the data point in the lower right corner of Figure 2. If this atypical program is removed, the R^2 ^for the remaining Indian sites is 0.72.

### Sexually Transmitted Infection programs

**Figure 3:**
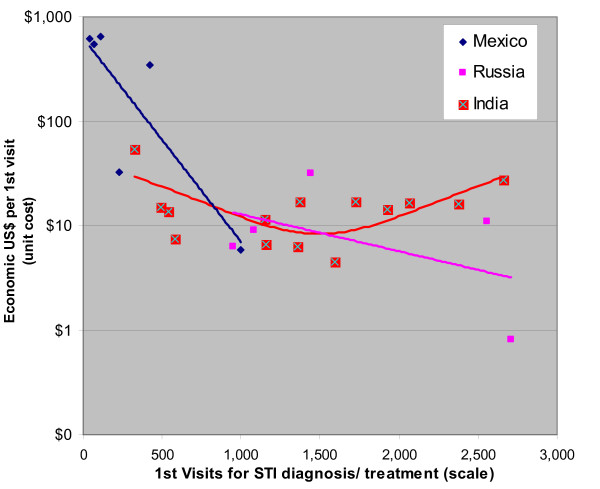


For sexually transmitted infection (STI) programs, data from three countries show at least modest scale effects over a smaller range in scale (See Figure 3). Scale varies 50-fold within Mexico, and 5-fold within Russia and India. Efficiency (cost per first visit for suspected STI) varies 100-fold within Mexico and 10-fold within Russia and India. Over all three countries, this cost varies from $650 to $0.82 per first visit. The proportion of variation explained by scale also varies widely: 70% (Mexico), 26% (Russia), and 42% (India). For Mexico, each doubling in scale is associated with a 35% – 90% decrease in cost per first visit, depending on starting point. For Russia, there is a 58% decrease in cost per first visit for a doubling in scale. STI programs in India were one of two intervention-country pairs that exhibited an upturn in unit costs. After declining from $53.77 per first visit at a program with 324 first visits, to $6.31 per first visit at a program with 1,357 first visits, unit cost rose to $27.33 for a program with 2,664 first visits.

### Information, Education and Communication programs

**Figure 4:**
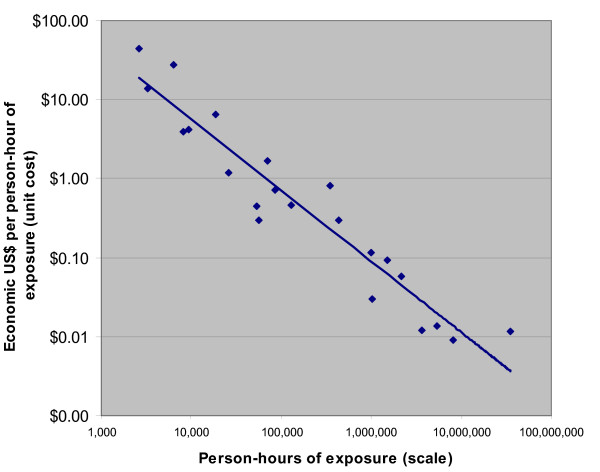


PANCEA data on information, education and communication (IEC) programs are restricted to Mexico. Scale varies widely: 10,000-fold, due to some programs having large electronic media components (See Figure 4). Efficiency (cost per hour of media contact) varies 5,000-fold. The proportion of variation explained by scale is 91%. A doubling in scale results in a 64% drop in cost per hour of contact and this association is highly statistically significant (p-value < 0.001).

### Risk reduction programs

Data on risk reduction program for injection drug users come only from Russia. Most programs focus on needle-syringe exchange. For these programs, scale varies 50-fold, and efficiency varies 40-fold. Scale explains 45% of variation in efficiency (cost per 50 needles exchanged). A doubling in scale is associated with a 34.5% reduction in cost per output (p-value = 0.004).

**Figure 5:**
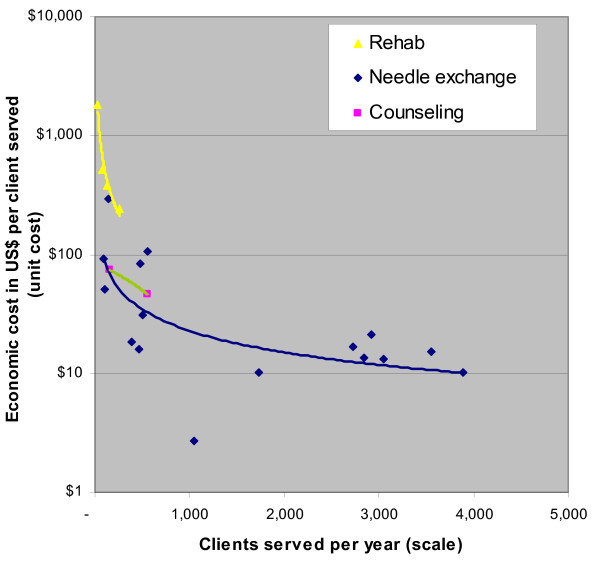


For inpatient rehabilitation programs (n = 4), scale explains 96% of variation in efficiency (cost per treatment episode). A doubling in scale is associated with a 47.9% reduction in cost per output (p-value = 0.022). The two programs that specialize in counseling also exhibit significant economies of scale, though this finding is weakly suggestive only because of the sample size (See Figure 5).

### Prevention of Mother to Child Transmission programs

**Figure 6:**
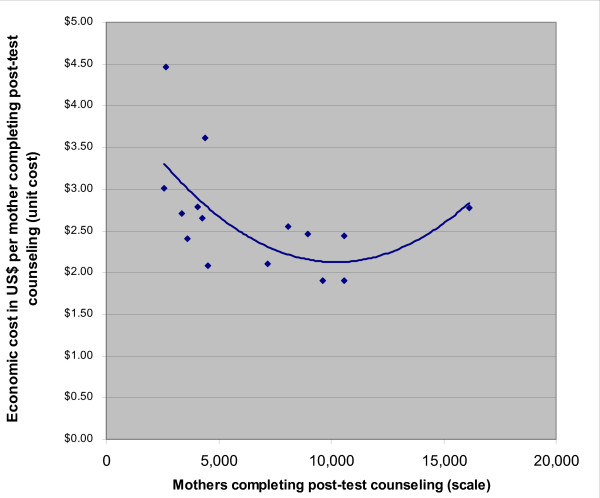


For prevention of mother-to-child HIV transmission programs (PMTCT), we report data only from India (other countries collected data from only one site each). For these programs, scale varies 6-fold, and efficiency varies 2-fold. Scale explains 42% of variation in efficiency (cost per mother completing post-test counseling). A doubling in scale from the first quartile output level is associated with a 23% reduction in cost per output. As shown in Figure 6, unit costs rise at output levels exceeding 10,000 women per year who receive post-test counseling. However, this up-turn in unit costs is due to only one data point. Without it, costs level off but do not increase. If the number of mother-neonate pairs receiving nevirapine is used as the output measure, we see strongly declining costs with scale up to 264 per year and no indication of an up-turn in unit costs (R^2 ^= 0.84) (Figure not shown).

## Discussion

With two exceptions, we found increasing efficiency across the full range of scale examined, in each of the 16 country-intervention pairs. Although the shape and strength of the association varied by country and intervention type, we found cost per key output and scale to be strongly and negatively correlated in almost all instances – with the fraction of variation in efficiency explained by scale ranging from 19% – 96%. Doubling in scale resulted in reductions in unit costs averaging 34.2% across all country-intervention pairs and ranging from 2.4% to 56%.

Over the scale that we examined, we saw no up-turn in cost per key output except for STI and PMTCT programs in India. The latter was due to only one data point (Figure 6). This suggests that for programs in similar demographic and epidemic settings one would expect to observe increasing efficiency at least up to similar levels of output. This is an encouraging finding as it suggests that the current global HIV prevention program will become less costly over a wide range of expansion. However, this finding must be tempered by the finding of an up-turn in unit cost reported in the previous study of SW programs in India [20], and by the up-turn reported here in PMTCT and STI programs. It must also be tempered by unpublished data from India by co-author Dandona using PANCEA methods for a more recent fiscal year that suggest an up-turn for VCT and SW programs. However, it is important to note that with simple linear functions, the regression trends were downward sloping in all cases.

There are a number of possible and non-mutually exclusive causes of the systematic economies of scale observed in the PANCEA data. One is that busier facilities are able to distribute their fixed costs over more cases, thus lowering their unit costs. A second is that busier facilities are better positioned to take advantage of potential lower prices through bulk purchases, sharing of services and other advantages of scale that lead to greater efficiency. Yet a third mechanism by which large scale may reduce unit costs is that program administrators learn how to integrate HIV prevention activities with routine services, reduce personnel "down-time," reduce supply loss and breakage, and generally deploy their resources more efficiently. This learning occurs over time and is thus an "economy of time" rather than of scale *per se*. However, in an era of program expansion, time and scale are highly correlated.

The first explanation, i.e., that unit costs decline as fixed costs are distributed over more output, is an arithmetic and programmatic truism, and therefore must contribute to the observed scale effects. The extent of that contribution depends on the portion that fixed costs constitute of total costs. The larger the fixed cost portion, the stronger the scale effect will be. Thus, it may be possible to increase efficiency by either increasing demand for services or finding ways to reduce costs that have been treated as fixed. Because inputs were market priced, we introduced a bias toward not being able to detect economies of scale arising from the ability to make bulk purchases. The third explanation, namely that program managers learn how to optimize deployment of the resources over time, and thus with scale, is supported by longitudinal analyses we conducted in PANCEA and other analyses we are conducting [11] (and unpublished data).

The purpose of the current analysis was to document the relationship between scale and unit cost and not to formally assess the relative contribution of each of these three causes. Our team is planning further analyses of the underlying determinants of efficiency using multivariate regression techniques to shed light on this question. We are currently analyzing longitudinal data on about 20% of the PANCEA sites and applying multivariate analyses to the existing data set in an effort to address these questions more definitively.

Our analysis has a number of important limitations. The data presented here are cross-sectional. Rather than documenting the change in unit costs over time within expanding programs, we tabulated the unit costs of many programs simultaneously and measured the association between scale and unit costs among them. Although these analyses are strongly suggestive of economies of scale within programs, they do not demonstrate it directly. In addition, this study does not use multivariate statistical methods to show the relative contribution of other possible predictors of unit costs, or to control for possible confounding factors. In particular, this paper does not assess the possible role of service quality as a mediator between unit cost and service volume. The effects of quality on unit costs are unclear. Higher quality services may require more resources and thus raise costs. It may also increase a programs reputation, thereby lowering outreach and promotion costs. By attracting more clients, it may also contribute to lowering unit costs via other economy of scale effects. We did not observe service delivery directly, and meaningful measures of quality vary by intervention type. However, for VCT programs, the largest program sub-set, we regressed cost per client on numerous indicators of program quality. We found no statistically significant associations between indicators of service quality and unit costs. For these reasons, it is conceivable, if unlikely, that the differences observed reflect program differences other than scale, but reflected in scale.

Because we did not observe an upturn in unit costs with larger scale for the vast majority of the programs, we are unable to address the question of what the optimal size of different types of prevention modalities might be. That is, we cannot identify optimal facility size, i.e., when it is more efficient to open a new facility in a nearby community rather than continue to expand services in an existing one. We believe that the lesson for now is, more services will usually reduce cost per service. Micro-economic theory suggests that an upturn in unit costs may be a signal to policy makers that the number of HIV prevention service providers should be increased in the relevant setting [13].

## Conclusion

The economies of scale data reported here may contribute to the ongoing effort to project the future cost of the global HIV prevention effort as it scales up. To date these efforts have been hampered by the inability to adjust costs by scale. For the five interventions for which we have a sufficient amount of data, the present findings may be able to provide useful guidance. As more PANCEA-type data are collected by us and by other research teams, it should be possible to add more points to this data set and thus arrive at increasingly precise estimates of the strength and range over which scale effects operate.

## Competing interests

The author(s) declare that they have no competing interests.

## Authors' contributions

All named authors were responsible for developing and integrating major elements of PANCEA methods, adapting those methods for application in their respective countries, and several contributed data for this paper Elliot Marseille lead the process of data analysis and interpretation, and the manuscript drafting and revision effort, with significant contributions from James G. Kahn, Lalit Dandona, Nell Marshall, Paul Gaist, Brandi Rollins, Mattias Lundberg, Sergio Bautista, Jo-Ann Du Plessis, and Mead Over. All authors read and approved the final manuscript.

## Pre-publication history

The pre-publication history for this paper can be accessed here:



## References

[B1] UNAIDS (2006). 2006 report on the global HIV/AIDS epidemic.

[B2] Kumaranayake L, Watts C (2000). Economic costs of HIV/AIDS prevention activities in sub-Saharan Africa. Aids.

[B3] Creese A, Floyd K, Alban A, Guinness L (2002). Cost-effectiveness of HIV/AIDS interventions in Africa: a systematic review of the evidence. Lancet.

[B4] Hogan DR, Baltussen R, Hayashi C, Lauer JA, Salomon JA (2005). Cost effectiveness analysis of strategies to combat HIV/AIDS in developing countries. Bmj.

[B5] Sweat M, Gregorich S, Sangiwa G, Furlonge C, Balmer D, Kamenga C, Grinstead O, Coates T (2000). Cost-effectiveness of voluntary HIV-1 counselling and testing in reducing sexual transmission of HIV-1 in Kenya and Tanzania [see comments]. Lancet.

[B6] Marseille E, Kahn JG, Mmiro F, Guay L, Musoke P, Fowler MG, Jackson JB (1999). Cost effectiveness of single-dose nevirapine regimen for mothers and babies to decrease vertical HIV-1 transmission in sub-Saharan Africa. Lancet.

[B7] Kahn JG, Marseille E, Auvert B (2006). Cost-effectiveness of male circumcision for HIV prevention in a South African setting. PLoS Med.

[B8] Dandona L, Sisodia P, Kumar SG, Ramesh YK, Kumar AA, Rao MC, Marseille E, Someshwar M, Marshall N, Kahn JG (2005). HIV prevention programmes for female sex workers in Andhra Pradesh, India: outputs, cost and efficiency. BMC Public Health.

[B9] Dandona L, Sisodia P, Prasad TL, Marseille E, Chalapathi Rao M, Kumar AA, Kumar SG, Ramesh YK, Over M, Someshwar M, Kahn JG (2005). Cost and efficiency of public sector sexually transmitted infection clinics in Andhra Pradesh, India. BMC Health Services Research.

[B10] Dandona L, Sisodia P, Ramesh YK, Kumar SGP, Kumar AA, Rao MC, Someshwar M, Hansl B, Marshall N, Marseille E, Kahn JG (2005). Cost and efficiency of HIV voluntary counselling and testing centres in Andhra Pradesh, India. Natl Med J India.

[B11] McConnel CE, Stanley N, du Plessis JA, Pitter CS, Abdulla F, Coovadia HM, Marseille E, Kahn JG (2005). The cost of a rapid-test VCT clinic in South Africa. S Afr Med J.

[B12] Soderlund N, Lavis J, Broomberg J, Mills A (1993). The costs of HIV prevention strategies in developing countries. Bull World Health Organ.

[B13] Henderson JM, Quandt RE (1971). Micro-Economic Theory: A Mathematical Approach.

[B14] McConnell JL (1945). Corporate Earnings by Size of Firm. Survey of Current Business.

[B15] Stigler JG (1958). The Economies of Scale. Journal of Law and Economics.

[B16] Over M (1986). The effect of scale on cost projections for a primary health care program in a developing country. Soc Sci Med.

[B17] Stover J, Bertozzi S, Gutierrez JP, Walker N, Stanecki KA, Greener R, Gouws E, Hankins C, Garnett GP, Salomon JA, Boerma JT, De Lay P, Ghys PD (2006). The Global Impact of Scaling-Up HIV/AIDS Prevention Programs in Low- and Middle-Income Countries. Science.

[B18] The World Bank, ACTafrica (2000). Costs of scaling HIV program activities to a national level in sub-Saharan Africa: Methods and Estimates.

[B19] Johns B, Baltussen R (2004). Accounting for the cost of scaling-up health interventions. Health Econ.

[B20] Guinness L, Kumaranayake L, Rajaraman B, Sankaranarayanan G, Vannela G, Raghupathi P, George A (2005). Does scale matter? The costs of HIV-prevention interventions for commercial sex workers in India. Bull World Health Organ.

[B21] Johns B, Torres TT (2005). Costs of scaling up health interventions: a systematic review. Health policy and planning.

[B22] Marseille E, Dandona L, Saba J, McConnel C, Rollins B, Gaist P, Lundberg M, Over M, Bertozzi S, Kahn JG (2004). Assessing the Efficiency of HIV Prevention around the World: Methods of the PANCEA Project. Health Serv Res.

